# Opposing roles of RNF8/RNF168 and deubiquitinating enzymes in ubiquitination-dependent DNA double-strand break response signaling and DNA-repair pathway choice

**DOI:** 10.1093/jrr/rrw027

**Published:** 2016-08-16

**Authors:** Shinichiro Nakada

**Affiliations:** Department of Bioregulation and Cellular Response, Graduate School of Medicine, Osaka University, Osaka, 565-0871, Japan

## Abstract

The E3 ubiquitin ligases ring finger protein (RNF) 8 and RNF168 transduce the DNA double-strand break (DSB) response (DDR) signal by ubiquitinating DSB sites. The depletion of RNF8 or RNF168 suppresses the accumulation of DNA-repair regulating factors such as 53BP1 and RAP80 at DSB sites, suggesting roles for RNF8- and RNF168-mediated ubiquitination in DSB repair. This mini-review provides a brief overview of the RNF8- and RNF168-dependent DDR-signaling and DNA-repair pathways. The choice of DNA-repair pathway when RNF8- and RNF168-mediated ubiquitination-dependent DDR signaling is negatively regulated by deubiquitinating enzymes (DUBs) is reviewed to clarify how the opposing roles of RNF8/RNF168 and DUBs regulate ubiquitination-dependent DDR signaling and the choice of DNA-repair pathway.

## INTRODUCTION

DNA double-strand breaks (DSBs) are the most deleterious type of DNA damage. DSBs must be detected and repaired immediately for cell survival. DSB repair pathways include error-free homologous recombination (HR) and error-prone non-homologous end joining (NHEJ). The optimum repair pathway may be actively selected to maintain genomic integrity. The most feasible mechanism for regulating the choice of DNA-repair pathway is DNA DSB response (DDR) signaling. The detection of DSBs by the MRE11•RAD50•NBS1 (MRN) complex initiates DDR signaling, which is transduced by Ser/Thr kinase ataxia telangiectasia mutated (ATM)-dependent phosphorylation and ring finger protein 8 (RNF8)- and RNF168-dependent ubiquitination, leading to the recruitment of the tumor protein p53 binding protein 1 (53BP1) and receptor-associated protein 80 (RAP80) to DSB sites. 53BP1 and RAP80 indirectly suppress HR and promote NHEJ. However, the relationship between RNF8- and RNF168-dependent DDR signaling and DNA-repair pathway choice remains to be elucidated. Several deubiquitinating enzymes (DUBs) have recently been identified as negative regulators of RNF8- and RNF168-dependent DDR signaling, and the depletion of some of these DUBs results in biased DNA-repair pathway choice. Accumulating evidence suggests a model in which the opposing roles of RNF8/RNF168 and DUBs in ubiquitination-dependent DDR signaling support the choice of DNA-repair pathway.

## DNA-REPAIR PATHWAY

DSBs are mainly repaired by NHEJ and HR. NHEJ directly ligates the DSB ends. If the overhangs of the DSB ends are compatible or the ends of the DSBs are blunt without other lesions present, the ends can be ligated without loss of nucleotides. When the overhangs of the DSB ends are incompatible or the ends of the DSBs have associated lesions, the ends of the DSBs are processed by nucleases before ligation. Subsequent DNA repair by NHEJ can lead to nucleotide loss [1, 2], and thus NHEJ is generally considered to be an error-prone DSB repair pathway. Upon the generation of DSBs, the Ku 70/80 heterodimer binds to the DSB end and protects it from degradation. The Ku 70/80 heterodimer recruits the DNA-dependent protein kinase (DNA-PK) catalytic subunit (DNA-PKcs). DNA-PKcs undergoes autophosphorylation, and DNA-PKcs subsequently phosphorylate other NHEJ component proteins such as Artemis [3]. X-ray repair cross-complementing protein 4 (XRCC4), XRCC4-like factor (XLF) and paralog of XRCC4 and XLF (PAXX) align DSB ends for efficient ligation, and ligase IV ligates the DSB ends [3–7]. HR restores the lost DNA sequence at DSB sites, using an undamaged DNA sequence on the identical sister chromatid as a template [8]. During the first stage of HR, the DSB ends are processed, thus generating a 3′-single strand DNA (ssDNA) overhang. This process, also called DNA end resection, is initiated by Mre11 in the MRN complex and/or by CtBP-interacting protein (CtIP) [9, 10]. The ssDNA overhang is rapidly bound by replication protein A (RPA), which is thought to remove secondary structures and protect the ssDNA from degradation [11]. Breast cancer 2 (BRCA2) facilitates the replacement of RPA with RAD51, and the resultant RAD51-ssDNA filament searches for a homologous DNA sequence on the identical sister chromatid. The RAD51-ssDNA filaments then invade the identical sister chromatid and anneal to the complementary ssDNA. DNA polymerases synthesize DNA by using the undamaged DNA strand template [8]. Thus, HR repairs DSBs without nucleotide deletion or alteration. Although HR and NHEJ are the main DSB repair pathways, additional mechanisms of DSB repair include alternative NHEJ (in which DNA ends are resected and repaired in a Ku 70/80-, ligase IV-, XRCC4- and XLF-independent manner) and other non-canonical repair pathways [3].

Because HR requires sister chromatids, HR is restricted only to late S and G_2_ phases of the cell cycle. In contrast to HR, NHEJ repairs DSBs throughout the cell cycle. That is, both HR and NHEJ are available in late S and G_2_ phases. How do cells choose the DNA-repair pathway in G_2_ phase? Recent studies have provided clues to answer this question.

## DNA-REPAIR PATHWAY CHOICE

**Fig. 1.**
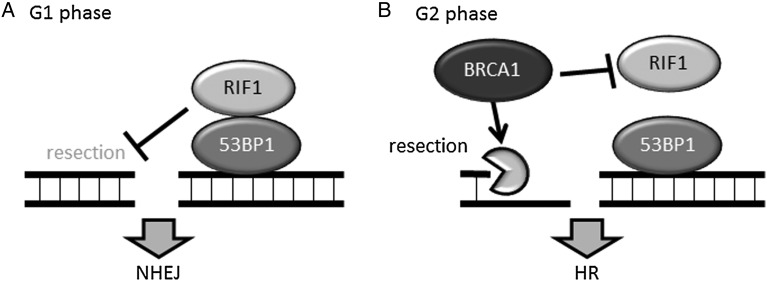
The 53BP1•RIF1 complex suppresses HR, and BRCA1 promotes HR. (A) The 53BP1**•**RIF1 complex suppresses DNA end resection in G_1_ phase. (B) BRCA1 inhibits RIF1 accumulation at DSB sites and enables DNA end resection in G_2_ phase.

In response to DSBs, various molecules are recruited to DSB sites. The accumulation of these molecules at DSB sites is clearly visible as foci (generally called **i**onizing **r**adiation **i**nduced **f**oci: IRIF) in the nucleus through immunofluorescence microscopy. 53BP1 and breast cancer 1 (BRCA1) are the best-known molecules that form foci at DSB sites. 53BP1 is considered to be an NHEJ-promoting protein because 53BP1-null cells exhibit ionizing radiation (IR) sensitivity, and 53BP1 knockout mice exhibit abnormalities in V(D)J recombination and class switch recombination [12, 13]. Several groups have recently revealed that the ATM-mediated phosphorylation of 53BP1 recruits Rap1 interacting factor 1 (RIF1) and PAX transcription activation domain interacting protein (PTIP); these proteins promote NHEJ by blocking DNA end resection in G_1_ phase cells [14–18] (Fig. 1A). In contrast to the 53BP1•RIF1 complex, BRCA1 promotes DNA end resection by recruiting activated CtIP to DSB sites [8, 19]. BRCA1 also recruits PALB2 and BRCA2, thereby facilitating RPA-RAD51 exchange on ssDNA [20]. In addition, BRCA1 inhibits RIF1 recruitment to DSB sites, releases the blockade of DNA end resection and promotes HR in G_2_ phase cells [14–17] (Fig. 1B). Thus, BRCA1 promotes HR. Although it is not clear whether the 53BP1•RIF1 complex physiologically inhibits BRCA1 accumulation at DSB sites and promotes NHEJ in G_2_ phase, an attractive model is that the 53BP1•RIF1 complex promotes NHEJ in the early stages of DDR, and the removal of RIF1 or the 53BP1•RIF1 complex by BRCA1 promotes HR in the late stages of DDR in G_2_ phase cells.

**Fig. 2.**
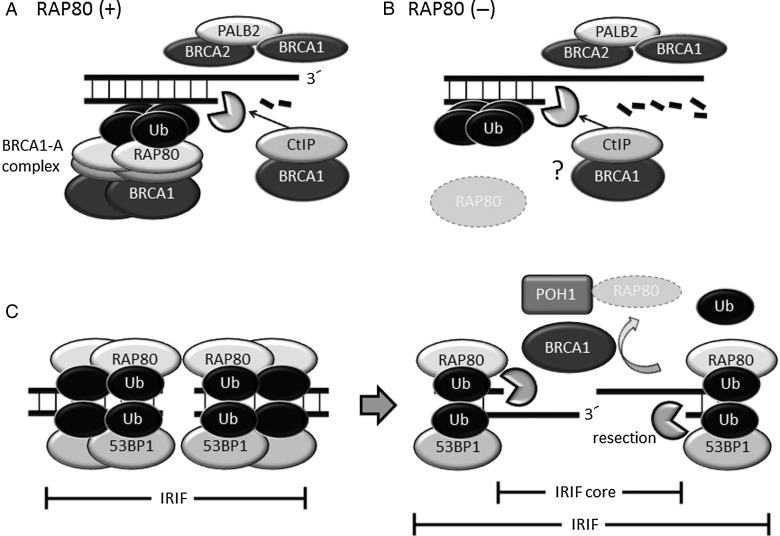
BRCA1 promotes DNA end resection, but the BRCA1-A complex suppresses excessive DNA end resection. (A) RAP80 binds to the K63-linked ubiquitin chain, forms the BRCA1-A complex and sequesters BRCA1 from DSB ends, enabling the suppression of excessive DNA end resection. The BRCA1**•**CtIP complex and BRCA1**•**PALB2**•**BRCA2 complex promote HR. (B) In the absence of RAP80, BRCA1 localizes to DSB sites independently of RAP80 and extensively promotes DNA end resection and HR. (C) BRCA1 and POH1 remove RAP80, ubiquitin chain and 53BP1 from the IRIF core, enabling DNA end resection. Ub: ubiquitin.

BRCA1 also forms a complex with RAP80, Abraxas, BRCA1/BRCA2-containing complex subunit 36 (BRCC36), BRCC45 and mediator of RAP80 interactions and targeting subunit of 40 kDa (MERIT40) [21–26]. This multiprotein complex is called the BRCA1-A complex. Many radiation-induced BRCA1 foci are considered to belong to the BRCA1-A complex (Fig. 2A) because the depletion of RAP80 results in significantly diminished formation of BRCA1 foci [27]. Although RAP80 plays a major role in recruiting BRCA1 to DSB sites, RAP80-depleted cells exhibit over-resection, increased HR activity and inefficient NHEJ [27, 28]. The enhanced HR in RAP80-depleted cells is cancelled completely by the depletion of BRCA1 [27], suggesting that BRCA1 promotes HR independent of RAP80, and RAP80 suppresses the HR-promoting function of BRCA1 (Fig. 2B). A recent study has revealed that RAP80 recruits BRCA1 to DSB-surrounding regions but not to DSB sites (Fig. 2A) [29]. This evidence strongly suggests that RAP80 sequesters BRCA1 from the edge of DSBs and fine-tunes the HR-promoting function of BRCA1. However, BRCA1 indirectly removes RAP80 and 53BP1 from the core of IRIF in G2 phase cells in the late stages of DDR [30] (details are described later). The absence of 53BP1 and RAP80 permits DNA end resection and RPA localization at the IRIF core (Fig. 2C). Thus, competition between BRCA1 and RAP80 also affects DNA-repair pathway choice.

## RNF8- AND RNF168-DEPENDENT DDR SIGNALING FACILITATES THE RECRUITMENT OF 53BP1 AND RAP80 TO DSB SITES

**Fig. 3.**
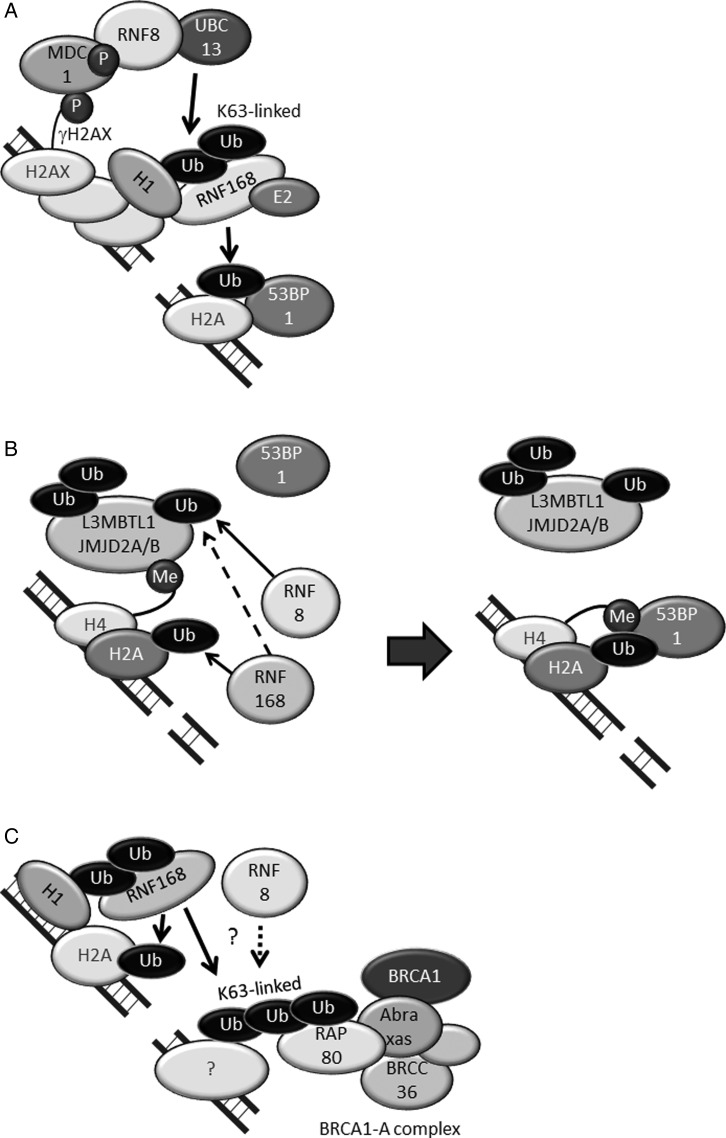
RNF8 and RNF168 promote the accumulation of 53BP1 and RAP80 at DSB sites. (A) RNF8 conjugates the K63-linked ubiquitin chain on histone H1 with UBC13. RNF168 accumulates at DSB sites by binding to K63-ubiquitinated histone H1 and then ubiquitinates histone H2A on K15 (H2AK15Ub). (B) 53BP1 interacts with H2AK15Ub and K20-dimethylated histone H4 (H4K20diMe). L3MBTL1, JMJD2A and JMJD2B are ubiquitinated and removed from DSB sites before 53BP1 accumulation. (C) RAP80, a component of the BRCA1-A complex, interacts with the K63-linked ubiquitin chain at DSB sites. Ub: ubiquitin, P: phosphate, Me: methyl.

The signaling cascade from the detection of DSBs to the accumulation of 53BP1 and RAP80 has been well studied (Fig. 3). Upon the generation of DSBs, the ends of the DSBs are detected by the MRN complex, which triggers the activation of ATM [31]. Subsequently, ATM phosphorylates histone H2AX in the region surrounding the DSBs, thus forming γH2AX. The mediator of DNA damage-checkpoint 1 (MDC1) then localizes to DSB sites by binding to γH2AX and is phosphorylated by ATM [32]. The phosphorylation of MDC1 promotes the recruitment of RNF8, and RNF8, in conjunction with the E2 conjugating enzyme UBC13, adds a lysine (K) 63-linked ubiquitin chain to histone H1; this chain serves as a scaffold for recruitment of ubiquitin binding proteins and does not induce protein degradation [33] (Fig. 3A) [34–39]. RNF168 then interacts with the K63-linked ubiquitin chain conjugated on ubiquitinated H1 through its ubiquitination-dependent DSB recruitment module 1 (UDM1), which consists of LR motif 1 (LRM1), UIM- and MIU-related UBD (UMI) [40], and motif interacting with ubiquitin 1 (MIU1) (Fig. 3A) [39, 41]. After binding to the ubiquitin chain, RNF168 ubiquitinates histone H2A on K15 (H2AK15Ub) at DSB sites, and 53BP1 interacts with H2AK15Ub through its ubiquitination-dependent recruitment (UDR) motif (Fig. 3A) [42]. RNF168 itself also interacts with ubiquitinated histone H2A through UDM2, which consists of MIU2 and LRM2, thus amplifying the ubiquitination-dependent DDR [39, 41]. Although histone H2A ubiquitination on K15 is mediated by UBCH5a or UBCH5c and not UBC13 *in vitro* [42, 43], 53BP1 forms foci at DSB sites when all UBE2D family E2-conjugating enzymes (UBCH5a, UBCH5b, UBCH5c and UBCH5d) are simultaneously depleted by siRNA [44]. Therefore, the E2 that conjugates ubiquitin on K15 of histone H2A *in vivo* remains to be elucidated.

For the retention of 53BP1 at DSB sites, an interaction between the Tudor domain of 53BP1 and K20-dimethylated histone H4 (H4K20Me2) is required (Fig. 3B) [45]. H4K20me2 is abundant in normal nuclei but is constitutively masked by the polycomb molecule L3MBTL1 [46] and the demethylases JMJD2A and JMJD2B [47]. At DSB sites, these proteins are ubiquitinated by RNF8 and RNF168, and removed from chromatin in a valosin-containing protein (VCP)/p97-dependent manner. Non-K63-linked ubiquitination is required [46–48]. After these proteins are removed from DSB sites, 53BP1 binds to the exposed H4K20me2 through its Tudor domain (Fig. 3B).

RAP80, a component of the BRCA1-A complex, has tandem ubiquitin-interacting motifs (UIMs) that enable specific binding to the K63-linked ubiquitin chain [22, 23, 49]. It is unclear whether RNF168 synthesizes K63-linked ubiquitin chains with UBC13 [39], but the recruitment of RAP80 to DSB sites depends on RNF168 (Fig. 3C) [37]. The K63-linked ubiquitinated protein to which RAP80 binds has not been identified.

The evidence described above suggests that the RNF8- and RNF168-dependent DDR signal suppresses HR and promotes NHEJ by recruiting 53BP1 and RAP80.

## SUPPRESSION OF RNF8- AND RNF168-DEPENDENT DDR BY DUBS AFFECTS THE CHOICE OF DNA-REPAIR PATHWAY

**Fig. 4.**
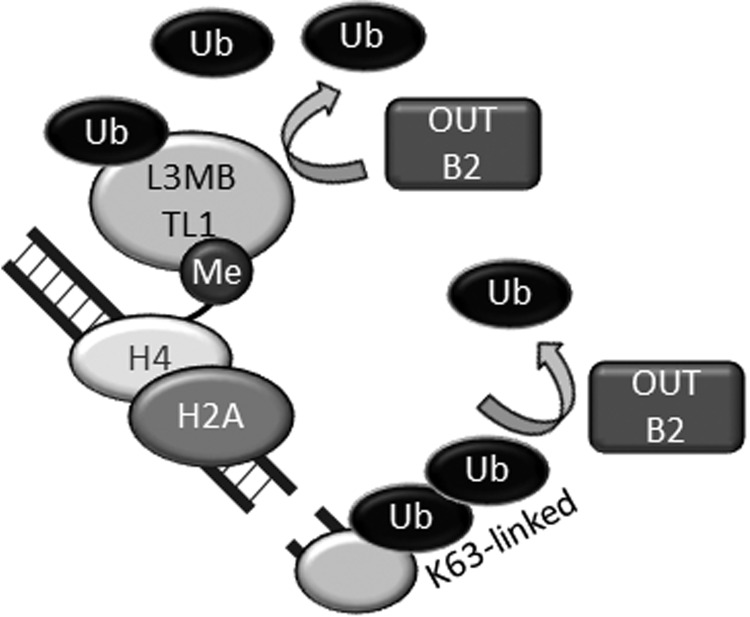
OTUB2 suppresses RNF8-dependent ubiquitination. OTUB2 deubiquitinates K63-linked ubiquitin chain synthesized by RNF8-UBC13 and suppresses excessive accumulation of RNF168 at DSB sites. OTUB2 also deubiquitinates L3MBTL1, properly maintains it on chromatin and suppresses the excessive accumulation of 53BP1. Ub: ubiquitin, Me: methyl.

**Fig. 5.**
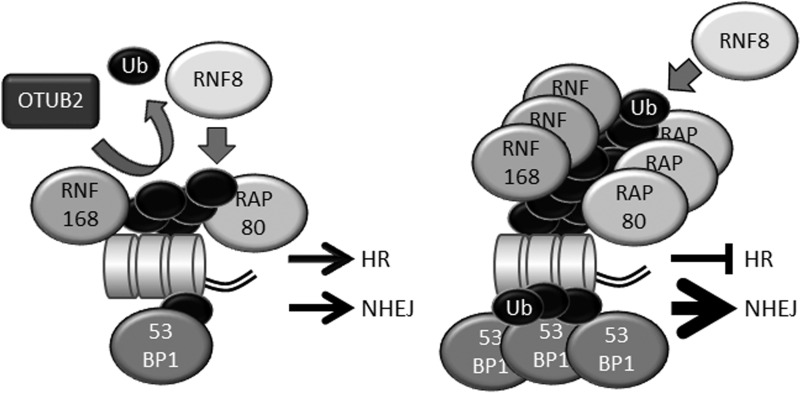
The role of the opposing activities of RNF8-RNF168 (ubiquitination) and OTUB2 (deubiquitination). (A) OTUB2 fine-tunes RNF8-dependent ubiquitination and suppresses the recruitment of excessive RNF168, RAP80 and 53BP1 to DSB sites, enabling the proper choice of DNA-repair pathway. (B) Accelerated RNF8-dependent ubiquitination results in excessive accumulation of RNF168, RAP80 and 53BP1, in turn promoting NHEJ and suppressing HR. Ub: ubiquitin.

In phosphorylation-dependent DDR signaling, phosphatases counteract ATM-dependent phosphorylation. For example, protein phosphatases PP4 and PP2A dephosphorylate γ-H2AX [50–52]. Similarly, DUBs counteract ubiquitination-dependent DDR signaling [53]. OTU deubiquitinase, ubiquitin aldehyde binding 2 (OTUB2) is an OTU family DUB that is involved in the RNF8- and RNF168-dependent DDR. OTUB2 does not exhibit strong linkage specificity but efficiently cleaves K63-, K48- and K11-linked ubiquitin chains [54]. In cells, OTUB2 suppresses the recruitment of RNF168 to DSB sites in a DUB activity-dependent manner. OTUB2 also deubiquitinates L3MBTL1 *in vivo* and *in vitro* [48]*.* Thus, OTUB2 counteracts RNF8 (Fig. 4). However, OTUB2 does not suppress histone ubiquitination induced by RNF168 overexpression [48]. (Notably, overexpressed RNF168 can bypass RNF8 and induce core histone ubiquitination in the absence of RNF8 [37]. Therefore, overexpressed RNF168 can ubiquitinate core histones in OTUB2-overexpressing cells in which RNF8-dependent ubiquitination is strongly suppressed [48].) In OTUB2-depleted cells, the conjugation of ubiquitin and accumulation of RNF168, 53BP1 and RAP80 at DSB sites are significantly accelerated during early phases of the DDR [48], and total DSB repair is upregulated. However, DNA end resection and HR are suppressed in OTUB2-depleted cells [48]. Thus, the RNF8-RNF168 axis primarily suppresses HR and promotes non-HR-type DSB repair, e.g. NHEJ, and OTUB2 enables the initiation of HR by suppressing the excessive accumulation of 53BP1 and RAP80 in an early phase of the DDR (Fig. 5).

Proteasome (prosome, macropain) 26S subunit, non-ATPase, 14 (PSMD14/POH1), a component of the proteasome, is a JAMM/MPN(+) DUB involved in DNA-repair pathway control. POH1 is recruited to the IRIF core in a BRCA1-dependent manner, and promotes the clearance of RAP80, the ubiquitin chain and 53BP1 from the IRIF core in G2 phase cells in the late stages of DDR [30]. The detailed molecular mechanism remains to be elucidated, but one proposed model is that POH1 degrades RAP80 and the loss of RAP80-dependent protection of the ubiquitin chain promotes the removal of ubiquitin, leading to the removal of 53BP1 from the core of IRIF [30]. RAP80 and ubiquitin chains persist at the IRIF core in POH1-depleted cells, but the ubiquitin chains are removed from the IRIF core when POH1 and RAP80 are simultaneously depleted [30], suggesting that DUBs but not POH1 degrade ubiquitin chains. However, it is also possible that POH1 and other DUBs redundantly cleave the ubiquitin chain [30, 55]. POH1 also plays a role in maintaining JMJD2A on chromatin, which suppresses 53BP1 recruitment to chromatin [55]. After RAP80 and 53BP1 have been cleared from the IRIF core, nucleases promote DNA end resection, allowing HR to proceed (Fig. 2C). Thus, POH1 relieves the barriers imposed by 53BP1 and RAP80 in the late stages of DDR and induces the switch from NHEJ to HR [30]. This IRIF core model is reasonable and attractive, but it should be noted that another group has reported that depletion of POH1 does not affect DNA end resection [55].

BRCA1/BRCA2-containing Complex Subunit 3 (BRCC3/BRCC36), a component of the BRCA1-A complex, is another JAMM/MPN(+) family DUB involved in RNF8-RNF168-dependent DDR signaling [26]. BRCC36 specifically cleaves the K63-linked ubiquitin chain on histone H2A [56], and BRCC36-depletion enhances 53BP1 IRIF in RNF8-depleted cells, indicating that BRCC36 and RNF8 play opposing roles in ubiquitination-mediated DDR [26]. Although the suppressive role of BRCC36 in RNF8-dependent DDR suggests that BRCC36 promotes HR, the depletion of BRCC36 increases HR efficiency [27]. This discrepancy is probably due to inefficient accumulation of the BRCA1-A complex at DSB sites in BRCC36-depleted cells [57]. However, the physiological role of BRCC36 in DNA-repair pathway choice remains to be elucidated.

**Fig. 6.**
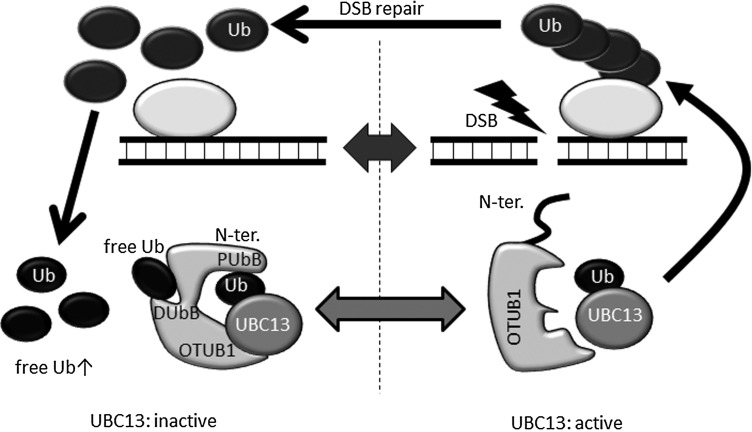
OTUB1 non-catalytically inhibits UBC13-dependent ubiquitination. OTUB1 interacts with ubiquitin-charged UBC13 (and UBE2D/2E family E2s) and inhibits E2-conjugating activity in a DUB activity-independent manner. A predicted model of OTUB1 action in DDR is shown. Deubiquitination of ubiquitinated proteins increases the local free ubiquitin concentration at DSB sites, enabling interaction with free ubiquitin and OTUB1. Free ubiquitin binding to the distal ubiquitin binding site of OTUB1 induces a conformational change of OTUB1 (see main text). The free ubiquitin-bound OTUB1 tightly binds to ubiquitin-charged UBC13 (and other E2 enzymes). The binding of OTUB1 to UBC13 (and other E2 enzymes) terminates ubiquitination-dependent DDR by inhibiting the activity of UBC13 (and other E2s). N-ter.: N-terminal residue, PUbB: proximal ubiquitin binding site, DUbB: distal ubiquitin binding site, Ub: ubiquitin.

Many other DUBs (e.g. OTUB1 [58, 59], ubiquitin specific peptidase 34 (USP34) [60] and USP44 [61]) have also been reported to be involved in RNF8-RNF168-dependent DDR. These DUBs are extensively reviewed in [53]. Among these DUBs, OTUB1 exhibits an interesting non-canonical function as a DUB. OTUB1 is an OTU family DUB specific for the K48-linked ubiquitin chain [62]. When OTUB1 cleaves the K48-linked chain, two ubiquitin binding sites in OTUB1 interact with both proximal and distal ubiquitins of the K48-linked ubiquitin chain [63]. One ubiquitin binding site that interacts with the proximal ubiquitin includes the ∼45 N-terminal residues of OTUB1 [63], and the other ubiquitin binding site that interacts with the distal ubiquitin includes the ∼190 C-terminal residue of OTUB1 [64]. The depletion of OTUB1 results in persistent ubiquitin chain formation at DSB sites, and the overexpression of OTUB1 inhibits 53BP1 IRIF, suggesting that OTUB1 is involved in RNF8- and RNF168-dependent DDR. However, the inhibitory effect of OTUB1 is independent of its DUB activity because the catalytically inactive mutant OTUB1^C91S^ suppresses 53BP1 IRIF and core histone ubiquitination as efficiently as wild-type OTUB1 [58]. How does OTUB1 inhibit ubiquitination-dependent DDR? Intriguingly, OTUB1 inhibits UBC13 and UBE2D/2E-family E2 conjugating enzymes in a DUB activity-independent manner [58]. For example, OTUB1 interacts with ubiquitin-charged UBC13 through its OTU domain and with ubiquitin conjugated on UBC13 through its N-terminal residue, and it suppresses the E2 activity of UBC13 physically but not enzymatically (Fig. 6) [58, 59, 65]. For activation of the inhibitory function, free ubiquitin must bind to the distal ubiquitin-binding site of OTUB1 [65]. Because the N-terminal residues are usually disordered, OTUB1 cannot interact with ubiquitin that is charged on UBC13. The binding of free ubiquitin to the distal ubiquitin binding site of OTUB1 triggers conformational changes in the OTU domain and the formation of a ubiquitin-binding helix in the N terminus of OTUB1, promoting tight interaction between OTUB1 and ubiquitin-charged UBC13 [65]. This mode of OTUB1 activity regulates DDR. This allosteric regulation of OTUB1 suggests that a locally highly elevated concentration of free ubiquitin, which can be produced by the deubiquitination of ubiquitinated proteins at DSB sites, promotes interaction between OTUB1 and ubiquitin-charged E2 conjugating enzymes. In this case, a physiological role of OTUB1 may be the termination of ubiquitination-dependent signaling at the end stage of DDR (Fig. 6).

## CONCLUSION

In conclusion, accumulating evidence suggests that the opposing functions of RNR8/RNF168 and DUBs affect the choice of DNA-repair pathway. Timely ubiquitination and the fine tuning of RNF8- and RNF168-dependent ubiquitination are probably keys for the appropriate choice of DNA-repair pathway. Some questions remain to be answered. How are the enzymatic activities of RNF8, RNF168 and DUBs regulated in the local area surrounding DSBs? What regulates the balance of ubiquitination and deubiquitination? Are there cell cycle-specific or DSB structure-specific regulations? Answering these questions will reveal the fundamental regulatory mechanism of DNA-repair pathway choice.

## FUNDING

This work was supported by KAKENHI 15H01183, JSPS KAKENHI 26241014; The Sumitomo Foundation; The Naito Foundation; and The Mochida Foundation.
